# Clinical profile and immediate outcomes of concurrent chemoradiation for cervical cancer at the Bugando medical centre in Mwanza, Tanzania

**DOI:** 10.4314/ahs.v25i2.27

**Published:** 2025-06

**Authors:** Aziza Ali Haji, Edgar Ndaboine, Beda Likonda, Oscar Ottoman, Richard Kiritta, Dismas Matovelo, Richard Rumanyika, Peter Rambau

**Affiliations:** 1 Department of Obstetrics and Gynecology, Weill Bugando School of Medicine, Catholic University of Health and Allied Sciences, P.O. Box 1464, Mwanza, Tanzania; 2 Department of Oncology, Weill Bugando School of Medicine, Catholic University of Health and Allied Sciences, P.O. Box 1464, Mwanza, Tanzania; 3 Department of Pathology, Weill Bugando School of Medicine, Catholic University of Health and Allied Sciences, P.O. Box 1464, Mwanza, Tanzania

**Keywords:** Cervical cancer, chemoradiation, toxicity, treatment outcomes, Tanzania

## Abstract

**Background:**

Globally, cervical cancer poses a challenge to public health. It is the fourth most prevalent cancer diagnosed in women worldwide, with an estimated annual death rate of 311,000. It is currently the most prevalent malignant disease in Tanzania in which the majority of patients with advanced cervical cancer have been offered concurrent chemoradiation (CCR). However, neither the clinical profile nor the immediate outcomes of these patients treated at the Bugando Medical Centre (BMC) have been thoroughly studied.

**Methodology:**

The prospective cohort study was conducted from November 2021 to April 2022, involving 160 eligible patients with histopathologically confirmed cervical cancer who received concurrent chemoradiation at BMC. Patients were followed for seven weeks, with the main clinical profiles of interest being age, histological type, histological tumor grade, FIGO disease stage, and HIV status, and the outcomes of interest being short-term clinical treatment-related toxicity and symptoms disappearance. The history and physical examination provided information about the patient's characteristics. Multivariate Logistic regression analysis was performed to evaluate the strength of the association between the patient's clinical profile and the short-term clinical treatment-related toxicity and symptoms disappearance. P-values less than 0.05 were considered statistically significant.

**Results:**

A total of 160 cervical cancer patients eligible were enrolled, with a median age of 50 years, 117 (73.5%) living in rural areas, and 152 (95%) being illiterate or having only primary education. The most common presenting symptoms were abnormal vaginal discharge 116 (72.5%) and bleeding 111 (69.4%). 119 (74%) patients presented at a late stage (IIB-1VA), 59 (36.9%) were HIV-positive, the majority 134 (83.7%) had squamous cell carcinoma, and 91 (56,2%) had a tumor of grade II type. At week 7, 60% of patients had a complete response to treatment. Vaginal bleeding and discharge improved, with only 12.5% and 6% of women still experiencing these symptoms respectively by week 7 however, 31% of reported cases of low back pain persisted. The majority of patients experienced tolerable grade II toxicities, including diarrhea (58%), vomiting (44.3%), and skin desquamation (52.5%). Fewer study participants reported grade III toxicity, and neither grade IV toxicity nor toxicity-related deaths were reported. Age > 60 years of age (OR 5.58; 95%CI 1.91-16.30; p = 0.002), late tumor stage at presentation (OR 3.36; 95%CI 1.53-7.37; p = 0.002), and HIV seropositivity (OR 11.8; 95%CI 4.87-28.6; p = 0.001) were associated with poor treatment responses.

**Conclusion:**

Cervical cancer still affects the majority of middle-aged women from rural areas with low levels of education and the majority present at an advanced stage. At BMC, concurrent chemoradiation has tolerable toxicity and a promising outcome. Early treatment outcomes are heavily influenced by HIV seropositivity, disease stage, and advanced age.

## Introduction

Globally, cervical cancer poses a challenge to public health. It is the fourth most prevalent cancer among women worldwide, with over 570,000 new cases and an estimated 311,000 deaths each year. While the incidence and mortality rates of cervical cancer are decreasing in high-income countries (HICs), they are increasing in low- and middle-income countries (LMICs). Approximately 80% of cervical cancer cases and 90% of deaths occur in low-income countries. Sub-Saharan Africa (SSA) has the highest number of cases and deaths^1^,^2^. According to 2018 data, Tanzania had the world's fourth-highest incidence rate of cervical cancer, with 59.1 new cases per 100,000 women (age-standardized to the world population). Cervical cancer mortality is also high, at 42.7 deaths per 100,000 people (age-standardized to the global population)^3^. Cervical cancer is the most common gynecological malignancy at Bugando Medical Centre (BMC), where approximately 400 patients are seen each year, contributing significantly to high morbidity and mortality^4^. Studies done in 2011 and 2014 at BMC found that up to 63.9% of patients had cervical cancer in stages IIB-IVA, and many of them also had HIV and anemia. However, these studies never looked at how well patients did after treatment^4^,^5^. The clinical stage of the disease at diagnosis often determines the prognosis and survival rate of a patient with cervical cancer, with the best outcomes seen in patients diagnosed at an early stage.

However, the outcome of treatment of these patients in LMICs has been poor because the majority of these patients present late to the hospital with advanced stage^6^. Cervical cancer is one of the preventable, early-detectable, and surgically or chemoradiation-treatable gynecological cancers. If resources for screening, early diagnosis, and potentially multiple treatment modalities are available, all of the aforementioned outcomes are feasible. The clinical problem is exacerbated by the country's lack of resources and poverty, sporadic cervical cancer screening, and high rates of HIV co-infection. Tanzania's government has prioritized addressing cervical cancer by opening a second treatment center in Mwanza. The second radiation center since 2017 follows the only one existing at Ocean Road Cancer Institute (ORCI) in Dar Es Salaam, 1200km from Mwanza^7^. Since the establishment of radiation services at BMC utilizing Cobalt 60 and brachytherapy, there has been no review of patients in terms of early outcomes such as toxicity and disappearance of symptoms, in addition to a review of their clinical profiles. According to studies, the response to cervical cancer treatment is primarily influenced by patient clinical characteristics such as age, FIGO clinical disease stage, HIV status, and treatment expertise. Knowing the clinical profile of patients who have been treated with chemoradiation for cervical cancer will affect how we care for these patients and how we modify policies for cervical cancer prevention. Treatment depends on the disease extent at diagnosis and locally available resources and might involve a radical hysterectomy, chemotherapy, or a combination of both. Advances in radiotherapy technology, like intensity-modulated radiotherapy, have made it so that women with locally advanced diseases have fewer side effects from their treatment^8^. A review of concurrent chemoradiation in patients with locally invasive cervical cancer is an informative way to examinehe patient management expertise provided. The majority of studies examining how patients fare after chemoradiation have been conducted in well-established centers with improved expertise, experience, and cutting-edge technology that influence patient preparation, staging, and treatment. Unfortunately, these results have been used to generalize the clinical outcomes of patients, even in places with few resources.

## Materials and methods

This was a prospective cohort study conducted at BMC from November 2021 to April 2022 BMC Oncology department involving 160 study participants who met the inclusion criteria. The study examines the pretreatment clinical profile of interest (age, FIGO disease stage, HIV status, histological type, and tumor grade) and the outcomes of interest (short-term clinical treatment-related toxicity and symptom disappearance). Histological confirmation of cervical cancer and a locally advanced tumor (FIGO stage IB1-IVA) planned for concurrent chemoradiation were the entry criteria. Following their assessment, they were informed about the study and voluntarily consented to participate. All patients adhere to concurrent chemoradiation hospital treatment guidelines, which include histological confirmation of cancer, Hemoglobin of 9.0gm/dl and above, normal renal and liver function tests, and clinical radiological staging. The radiation regimen includes at least 50 Gy of external beam radiation (EBRT) delivered Monday through Friday (5 days a week) for 5 weeks (25 fractions/session), followed by 3 sessions of 8 Gy/week of brachytherapy (2 weeks). Concurrent chemotherapy included one to six weekly cycles of Cisplatin 40 mg/m^2^, which were given to the patient on Mondays. The criteria for excluding patients were failing to consent, [EN1] being lost to follow-up, receiving palliative radiotherapy, and receiving adjuvant chemotherapy. To gather information, a history was taken and a physical examination was performed. The staging of cervical cancer was based on clinical and radiological data. Throughout the treatment, symptoms, and toxicity were assessed weekly, and toxicity was graded using the Common Terminology Criteria for Adverse Events (CTCEA criteria version 5). CTCEA - Grade denotes the seriousness of an adverse event (AE)^9^. The CTCAE lists Grades 1 through 5 and clinical descriptions of each AE. Grade 1: mild symptoms that do not necessitate treatment grade 2: moderate or minimal local or noninvasive intervention. Grade 3: Severe or medically significant, but not immediately life-threatening; hospitalization or prolongation is advised, with incapacitating and limiting self-care. Grade 4: life-threatening consequences, the indication of immediate intervention, and Grade 5: AE-related death. A pre-coded, structured questionnaire was used to collect data. BMC is the only tertiary hospital around Lake Victoria Zone and a university teaching hospital for the Catholic University of Health and Allied Sciences (CUHAS).

There are a variety of treatment options available through the BMC Oncology division, including diagnostic screenings, radical surgical procedures, radiation therapy (both external beam and intracavitary radiation), and chemotherapy. At the moment, there are at disposal a high-dose-rate (HDR) brachytherapy machine, a C-ARM x-ray unit, a cobalt-60-unit machine, and a 2D simulator. Categorical variables were summarized into proportions and percentages, while numerical variables were in mean and standard deviation. The Chi-square and Fisher's exact tests of association were used to examine the relationship between the patient's characteristics and histopathological findings. Variables with a p-value of less than 0.05 were considered statistically significant.

## Results

### Recruitment of patients

**Figure 1:**
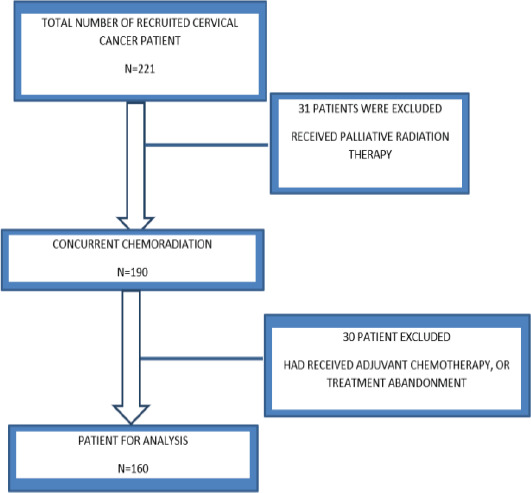
Requirement of patients

During the study period, 221 patients with a histological diagnosis of cervical cancer were managed; 160 of these patients met the study's inclusion criteria. (Figure 1).

### Clinical profile of cervical cancer patients treated at BMC with concurrent chemoradiation

The most striking findings of this cohort study were that the median age for cervical cancer was 50 years, 68% of cases were high parity (above para 4), 74% were at an advanced stage (stages IIB–IVA), illiteracy and low educational attainment accounted for 95% of all cases combined. 63% of the women in the study were HIV-positive, and abnormal vaginal discharge was the most common symptom they reported. Additional findings as shown in table 1.

**Table 1:** Clinical profile of cervical cancer patients treated at BMC with concurrent chemoradiation

Clinical profile	Number (n=160)	Percent (%)
**Age**		
≤ 40	31	19.4
41- 59	86	53.8
≥60	43	26.8
**Residency**		
Rural	117	73.5
Urban	43	26.5
**Marital status**		
Single	2	1.3
Divorce	33	20.6
Widow	32	20.0
Married	93	58.1
**Parity**		
Prime Para	4	2.5
Para 2-4	47	29.6
Para 5 and above	108	67.9
**Education Status**		
Illiterate	46	28.8
Primary	106	66.2
Secondary	5	3.1
College	3	1.9
**Job-status**		
Small scale farmers	146	91.3
Small scale merchants	14	8.7
**Symptoms**		
Abnormal vaginal bleeding	111	69.4
Abnormal vaginal discharge	116	72.5
Lower abdominal pain	92	57.5
Lower back pain	96	61.9
**Clinical staging**		
IB1 – IIA2	41	25.6
IIB - IVA	119	74.4
**Tumor grade**		
Grade 1	62	38.8
Grade 2	91	56.8
Grade 3	7	4.4
**Histological diagnosis**		
Squamous cell carcinoma	134	83.7
Adenocarcinoma	26	16.3
**HIV Group**		
HIV negative	101	63.1
HIV positive	59	36.9

### Overall treatment outcome according to the disappearance of symptoms

**Figure 2:**
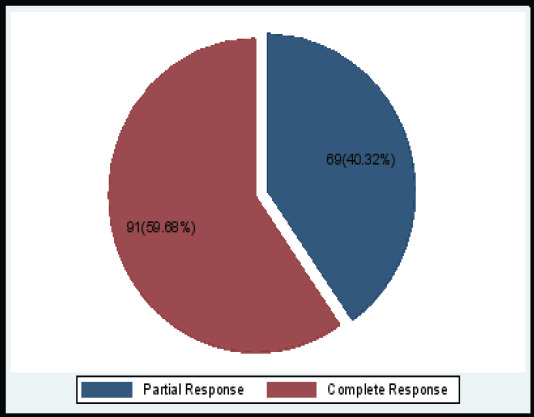
Distribution of population according to the disappearance of symptoms

Upon review of 160 patients seven weeks after CCR with EBRT, brachytherapy, and cisplatin, 60% had complete symptomatic remission and 40% had a partial response (Figure 2).

### Presence of symptoms weekly from Week 1 to Week 7 of concurrent chemoradiation treatment

Improvements were seen in all four major symptoms every week, with the greatest improvement seen in week 7. The greatest improvement was seen in vaginal bleeding and discharge, with only 12.5% and 6% of women still experiencing these symptoms by week 7. While 31% of reported cases of low back pain persisted at week 7 (Table 2).

**Table 2:** Weekly occurrence of symptoms in 160 (100%) study participants from Week 1 to Week 7 of chemo radiation treatment

	1^st^ week	2^nd^ week	3^rd^ week	4^th^ week	5^th^ week	6^th^ week	7^th^ week
**Abnormal vaginal discharge**	116 (72.5%)	100(62.5)	72(45.0)	58(36.3)	43(36.3)	30(18.8)	20(12.5)
**Abnormal vaginal bleeding**	111(69.3)	90(56.2)	60(25.0)	40(25.0)	32(20.0)	18(11.2)	10(6.25)
**Lower back pain**	96(60.0)	85(58.1)	73(45.6)	65(40.6)	42(26.2)	38(23.7)	50(31.2)
**Lower abdominal pain**	92(57.5)	82(51.2)	70(43.8)	62(38.7)	58(36.2)	42(26.2)	35(21.9)

### Adverse treatment effects due to treatment toxicity, as reported by patients every week

Toxicity was graded using CTCAE criteria. Overall, treatment-related toxicity was higher in the first two weeks of chemoradiation and significantly improved in the final four weeks. The majority of patients reported tolerable grade II toxicity symptoms, such as diarrhea (58%), vomiting (44.3%), and skin desquamation (52.5%). Fewer study participants reported grade III toxicity, and no grade IV or deaths were reported as a result of toxicity (Table 3).

**Table 3:** Adverse treatment effects due to treatment toxicity, as reported by patients every week

	Number(n)	Percent (%)	
**Toxicity grades** **Diarrhea**			
	No diarrhea	8	5.0
	Grade I	36	22.5
	Grade II	93	58.1
	Grade III	23	14.4
**Vomiting**			
	No vomiting	39	24.4
	Grade I	44	27.5
	Grade II	71	44.3
	Grade III	6	3.8
**Skin desquamation**			
	No skin changes	30	18.8
	Grade I	38	23.7
	Grade II	84	52.5
	Grade III	8	5.0
**Painful urination**			
	No toxicity	115	71.8
	Grade I	15	9.4
	Grade II	29	18.2
	Grade III	1	0.6

### Factors associated with the disappearance of symptoms

The disappearance of clinical symptoms after chemoradiation was influenced by patient age, FIGO disease stage, and HIV seropositivity. Age greater than 60 years (OR 5.58; 95%CI 1.91-16.30; p-value = 0.002), late tumor stage at presentation (OR 3.36; 95%CI 1.53-7.37; p-value = 0.002), and HIV seropositivity (OR 11.8; 95%CI 4.87-28.6; p-value = 0.001) were associated with the late disappearance of the symptoms. These factors were statistically significant in multivariate logistic regression analysis compared to the patient's clinical profile (Table 4).

**Table 4:** The relationship between treatment responses (symptoms disappearance) and the clinical profile of the patient at week 7 of follow-up

Clinico-pathological characteristics.		Treatment response		Bivariate		Multivariate Logisticregression	
		Complete response n(%)	Partial response n(%)	^χ2^	p-value	aOR(95% CI)	p-Value
**Age group(years)**							
	≤ 40	17(54.8)	14(45.2)			1	
	40-59	44(51.2)	42(48.8)	14.54	**0.001**	1.37(0.58 -3.23)	0.468
	≥ 60	8(18.6)	35(81.4)			5.58(1.91-16.30)	**0.002**
**Tumor stage**							
	Early (I-IIA2)	27(65.6)	14(34.2)	11.61	**0.001**	1	
	Late (IIB-IVA)	42(35.3)	77(64.7)			3.36(1.53-7.37)	**0.002**
**HIV group**							
	HIV negative	62(61.4)	39(38.6)	0.001	**0.001**	1	
	HIV positive	7(11.9)	52(88.9)			11.8(4.87-28.6)	**0.001**
**Histological Type**							
	SCC	62(46.3)	72(53.7)	0.068	0.052		
	Adenocarcinoma	7(26.9)	19(73.1)				
**Tumor grade**							
	Grade I	24(35.3)	38(41.3)	3.486	0.175		
	Grade II	43(65.3)	48(52.2)				
	Grade III	1(1.5)	6(4.4)				

## Discussion

This is the first study at BMC to demonstrate how the clinical profile of cervical cancer patients influences the early treatment outcome of concurrent chemoradiation (CCR). The majority of patients in this study were from rural areas, had low levels of education, were in their middle years, and 36.9% were HIV-positive. These results are consistent with those of other studies in Tanzania, which demonstrated that the natural history of cervical cancer continues to affect those with low socioeconomic status and those with compromised immune systems^4^,^5^. Access to screening in Tanzania is primarily restricted to urban areas, and the low education level of a significant portion of our community affects their comprehension, bringing with it other perceptions and beliefs, such as superstitions and bad luck^7^,^10^. This would explain why 73.5% of our study participants were from rural areas and less than 5% had a secondary or higher level of education. HIV is known to induce immunosuppression, which in turn modifies the natural history of HPV (a causative organism for cervical cancer). This increases the likelihood of persistent infection with high-risk HPV, which will eventually lead to cervical dysplasia and cancer^11^. To effectively combat cervical cancer, it is necessary to reduce the burden of HIV, expand access to screening in rural communities, and empower women by increasing literacy levels, at the very least by ensuring that a substantial number of girls attend school^7^,^12^.

This study also revealed that nearly three-quarters of the patients were in an advanced stage (stage IIB or higher); invasive cervical cancer at this stage is extremely difficult to treat. This stage indicates significant delays. Because patients typically experience vaginal discharge and bleeding for years before disease progression from the early stage to the advanced stage. The delays may be due to patient factors or the attending physician's failure to make an early diagnosis^7^. Studies conducted in Tanzania indicate that even practicing clinicians lack awareness. Women who present to hospitals with early symptoms of cervical cancer are frequently misdiagnosed as suffering from other sexually transmitted diseases. This is particularly prevalent in rural Tanzania, where the majority of patients are managed by clinical officers and nurses who lack proper cervical cancer training and expertise^13^,^14^.

In this study, the overall treatment efficacy, as measured by the disappearance of symptoms, was 60%. This is encouraging, as the disappearance of symptoms typically indicates an improved treatment response; however, the long-term impact of cancer clearance must be determined later. It has been demonstrated that CCR is more effective than radiotherapy alone in treating cervical cancer, even in its advanced stages, and increases cervical cancer patient survival. Multiple randomized controlled trials demonstrate a roughly 10% survival advantage at 5 years for radiation with concurrent platinum-based chemotherapy versus radiation alone in women with locally advanced invasive cervical cancer^15^,^16^.

The disappearance of symptoms and early clinical chemoradiation toxicity were the outcomes of interest in this study. The majority of the study population had vaginal bleeding and watery discharge, and as in previous studies, symptoms gradually disappeared after treatment in many cases^6^,^17^. The pain persisted in nearly half of the patients who presented with pain at week 7 of treatment, which could be explained by the fact that the majority of patients presented with pain at an advanced stage and that these symptoms would likely diminish over time. However, research indicates that pain is a common complication of cancer and its treatment and that it is frequently underreported and inadequately described^18^.

In this study, the majority of patients experienced mild to moderate toxicity. The most common grade 3 toxicity observed was gastrointestinal symptoms. Consequently, based on these findings, the majority of radiation toxicity symptoms in our setting were well-manageable during treatment with supportive therapy, and almost all types of toxicity observed in the study showed significant symptomatic improvement after treatment. Chemoradiation may be well tolerated by both elderly patients with cervical cancer and those with HIV seroconversion.

Thus, the findings of this study are consistent with those of other studies that compared radiation toxicity using modern conventional methods (3D) imaging with a Linarc machine (used in high-tech settings) and 2D imaging with a Cobalt machine (used in our setting)^19^-^21^. Our institution's experience with CCR for locally advanced cervical cancer demonstrated efficacy with tolerable toxicity. All 160 participants in the study completed the entircourse of treatment. During treatment, we did not observe any women with life-threatening adverse events or deaths. This has provided us with a better understanding of our performance and will serve as a foundation for future research examining the long-term benefits of chemoradiation. When we compared the patient's clinical profile to the symptomatic treatment response, we found that the patient's advanced age (above 60 years), HIV positivity, and advanced disease stage (IIB-IVA) were significantly associated with delayed or persistent symptoms after treatment. Multiple studies have reported similar results^6^,^22^,^23^. Given this, there is a need to increase HIV-positive women's awareness of cervical cancer. HIV screening and cervical cancer screening should occur simultaneously, and vice versa. In 2017, a mass cervical cancer screening campaign on the island of Ukerewe in Lake Victoria revealed that 80% of women did not know their HIV status^24^. If dual screening emphasis is not implemented, the majority of women will continue to suffer from both conditions. Our random screening program employs the see-and-treat Visual inspection with acetic acid (VIA) method, which is impractical for older women who require a pap test. VIA screening is unlikely to detect endocervical cancer, which accounts for the majority of cases and is therefore diagnosed at a late stage^7^,^25^.

## Limitations

It was difficult to distinguish between disease-induced and radiation-induced pain. The staging was determined solely by a physician's clinical evaluation and few radiological findings. Since the staging was performed by multiple specialists, this might not be accurate. Due to financial constraints, the duration of the follow-up was limited to seven weeks, which is insufficient for disease clearance.

## Conclusion

The majority of women who develop cervical cancer are in their middle ages, live in rural areas, have a low level of education, and typically display symptoms when the disease is already in the advanced stage. At BMC, concurrent chemoradiation has manageable toxicity and an outcome that shows promise. The presence of HIV, the advanced age of the patient, and the stage of the disease all have a significant impact on early treatment outcomes.

## Recommendation

This study revealed that cervical cancer remains a significant burden because the majority of patients present late, making it more difficult to treat; consequently, there is a need to raise awareness and strengthen preventive measures. Additional funding is necessary for studies with longer follow-up periods to describe survival and treatment outcomes at our center.

## Study strength

All participants in the study completed the required treatment on time, and no cases for follow-up were lost. The majority of the study's participants were from the Lake Zone, where the cervical cancer burden and clinical profile have been highlighted.

## Data Availability

The dataset used and/or analyzed during the current study is available from the corresponding author upon request.
